# Multilevel Determinants of Tuberculosis Treatment Interruption in Rural South Africa: Insights from Primary Healthcare Nurses

**DOI:** 10.3390/ijerph23050598

**Published:** 2026-05-01

**Authors:** Mlandeli Tsibiyane, Lindiwe Modest Faye, Kululwa Ndayi, Ncomeka Sineke, Londele Tyeshani, Monwabisi Faleni

**Affiliations:** 1 School of Laboratory Medicine and Pathology, Faculty of Medicine and Health Sciences, Walter Sisulu University, Mthatha 5099, South Africa; 207625743@mywsu.ac.za (M.T.); kndayi@wsu.ac.za (K.N.); 209101237@mywsu.ac.za (N.S.); ltyeshani@wsu.ac.za (L.T.); 2 School of Public Health, Faculty of Medicine and Health Sciences, Walter Sisulu University, Mthatha 5099, South Africa

**Keywords:** tuberculosis, MDR-TB, adherence, treatment, healthcare workers

## Abstract

**Highlights:**

**Public health relevance—How does this work relate to a public health issue?**

- The study focuses on tuberculosis (TB) treatment interruption, a persistent public health issue that undermines TB control efforts and contributes to continuous transmission in high-burden settings like South Africa.
- The study, which examines patient, social, and health system factors impacting treatment interruption in a rural community, provides context-specific evidence on hurdles to TB treatment adherence.

**Public health significance—Why is this work of significance to public health?**

- Treatment interruption increases the likelihood of poor clinical results, disease re-lapse, and the development of drug-resistant tuberculosis, posing a significant danger to TB elimination efforts.
- Understanding the causes of treatment interruption is crucial for developing tailored strategies to increase treatment success rates and reduce the burden of tuberculosis in vulnerable populations.

**Public health implications—What are the key implications or messages for practitioners, policy makers and/or researchers in public health?**

- The findings underline the importance of integrated tuberculosis programmes that address socioeconomic hurdles such as transportation costs, food insecurity, and a lack of social support in addition to clinical care.
- To increase adherence and treatment outcomes in high-burden settings, policymak-ers and program managers should strengthen community-based treatment support, patient tracking systems, and decentralised tuberculosis services.

**Abstract:**

Background: Tuberculosis (TB) remains a major public health challenge globally, particularly in high-burden countries such as South Africa. Treatment interruption is a critical barrier to effective TB control, contributing to poor treatment outcomes, increased risk of multidrug-resistant tuberculosis (MDR-TB), and continued community transmission. Understanding the determinants of treatment interruption in rural healthcare settings is essential for strengthening TB programme implementation. Methods: This qualitative study explored the factors influencing TB treatment interruption from the perspectives of professional nurses working in primary healthcare facilities in the Nyandeni Subdistrict, Eastern Cape, South Africa. Semi-structured interviews were conducted with nurses involved in TB programme implementation. Data were analysed using thematic analysis following the six-phase approach described by Braun and Clarke. Descriptive statistical analyses were also used to summarize participant characteristics, including age and years of nursing experience. Conceptual frameworks were developed to illustrate the multilevel determinants of TB treatment interruption. Results: Participants had a mean age of 40.6 years and an average of 14.2 years of nursing experience, reflecting a workforce with substantial clinical exposure to TB management. Thematic analysis identified multiple interconnected determinants of treatment interruption. Key barriers included poverty, food insecurity, transport costs, long distances to healthcare facilities, limited family support, and challenges related to patient tracing. These factors interact across structural, community, health system, and interpersonal levels to influence patient adherence behaviour. Conceptual models developed from the findings illustrate the complex pathways through which these determinants contribute to treatment interruption and programme-level consequences such as reduced treatment success and increased risk of MDR-TB. Conclusions: TB treatment interruption in rural settings is driven by multilevel socioeconomic and health system determinants rather than individual patient behaviour alone. Strengthening community health worker programmes, improving patient tracing systems, addressing socioeconomic barriers, and enhancing community-based support mechanisms are essential for improving treatment adherence. Integrated, multisectoral interventions are required to strengthen TB programme outcomes in rural high-burden settings.

## 1. Introduction

Tuberculosis (TB) remains one of the leading infectious causes of morbidity and mortality worldwide [1]. Despite significant global progress in TB control, the disease continues to disproportionately affect low- and middle-income countries, particularly in sub-Saharan Africa [2]. South Africa remains among the countries with the highest TB burden globally, with persistent challenges related to treatment adherence, drug resistance, and ongoing community transmission [3].

Successful TB control depends heavily on sustained treatment adherence [4]. Standard TB treatment regimens require patients to complete several months of continuous therapy, often under challenging socioeconomic conditions. When patients interrupt treatment, the consequences extend beyond individual health outcomes. Treatment interruption increases the risk of treatment failure, relapse, development of multidrug-resistant tuberculosis (MDR-TB), and continued transmission within communities [5]. Addressing the factors contributing to treatment interruption is therefore a critical priority for TB programmes.

In rural healthcare settings, barriers to TB treatment adherence are often complex and multifaceted. Patients frequently face socioeconomic challenges such as poverty, food insecurity, and unemployment, which may limit their ability to attend clinic appointments or tolerate medication [6,7]. Geographic barriers, including long distances to healthcare facilities and high transport costs, further constrain access to TB services. In addition, health system challenges such as staffing shortages, limited patient tracing capacity, and resource constraints may weaken adherence monitoring and follow-up mechanisms [8].

Social and household factors also influence treatment adherence. Disclosure of TB status, family support, and social stigma can significantly affect patients’ willingness and ability to continue treatment [9]. Patients who lack supportive social networks may experience greater difficulties managing the physical, emotional, and logistical demands of long-term TB treatment [10].

Healthcare providers working within primary healthcare facilities play a central role in managing TB treatment and monitoring patient adherence. Professional nurses are often responsible for initiating treatment, providing patient education, monitoring treatment progress, and coordinating patient tracing efforts when individuals miss appointments [11]. Their perspectives therefore provide valuable insights into the practical challenges faced in implementing TB programmes in rural settings.

Understanding healthcare providers’ experiences is particularly important for identifying systemic and contextual barriers that may not be fully captured through routine programme data. Qualitative research can provide in-depth insights into the complex interactions between socioeconomic conditions, health system capacity, and patient behaviour that influence treatment adherence [12].

The present study explores the factors influencing TB treatment interruption from the perspectives of professional nurses working in primary healthcare facilities in the Nyandeni Sub-district of the Eastern Cape Province, South Africa. By examining these perspectives, the study aims to identify the key determinants of treatment interruption and to develop conceptual frameworks that illustrate the multilevel pathways through which these determinants influence TB treatment adherence.

## 2. Materials and Methods

### 2.1. Study Design and Setting

This study used a qualitative exploratory methodology to evaluate healthcare personnel’ perspectives of factors influencing TB treatment interruption. A qualitative approach was deemed acceptable for gaining in-depth insights on the contextual, societal, and health-system-related problems influencing treatment adherence. The study was conducted out in six primary healthcare (PHC) facilities in the Nyandeni Subdistrict of the Eastern Cape Province, South Africa. This is a predominantly rural setting with a high TB burden, socioeconomic constraints, and limited access to healthcare services. PHC facilities in this scenario are the first point of contact for TB diagnosis, treatment initiation, and adherence monitoring.

The six PHC facilities included in this study were not exhaustive of all facilities within the Nyandeni Subdistrict but were purposively selected to ensure representation of diverse rural healthcare contexts. Facility selection was conducted in consultation with subdistrict health authorities and facility managers, based on predefined criteria including (i) active implementation of TB services, (ii) availability of professional nurses with direct involvement in TB patient management, and (iii) variation in geographic location and operational characteristics. This purposive approach allowed for the inclusion of facilities reflecting different service delivery contexts and challenges related to TB treatment adherence within the subdistrict.

### 2.2. Study Population and Sampling

Professional nurses involved in the execution of TB programmes at selected PHC institutions made up the study population. Nurses were eligible if they had direct experience with tuberculosis patient management, including therapy start, monitoring, and patient tracking. A purposive sample technique was employed to select participants who had relevant experience and knowledge of TB program operations. Facility administrators helped to identify qualified individuals. Sampling proceeded until data saturation, which was characterized as the lack of fresh themes or insights emerging from the interviews.

Professional nurses were recruited using a purposive sampling strategy within the selected primary healthcare facilities. Facility managers assisted in identifying nurses who met the inclusion criteria, specifically those directly involved in TB patient management, including treatment initiation, monitoring, and patient tracing. Eligible participants were approached by the research team, provided with detailed information about the study, and invited to participate on a voluntary basis. Sampling continued until data saturation was reached.

### 2.3. Data Collection

All interviews were conducted in person by the primary researcher, who was trained in qualitative research methods and had prior experience in conducting semi-structured interviews. Interviews were held in private rooms within the participating primary healthcare facilities to ensure confidentiality and minimize disruptions. A face-to-face approach was chosen to enable in-depth engagement, facilitate probing for detailed responses, and capture non-verbal cues relevant to participants’ experiences. No interviews were conducted via telephone or virtual platforms. The interviewer had no prior supervisory relationship with participants, reducing the risk of response bias.

Interviews were held in private rooms within healthcare facilities to maintain confidentiality and minimize disturbances. All interviews were performed in English or the individuals’ preferred language, with clarification and prodding to elicit thorough responses. Interviews were audio-recorded with the participants’ consent to ensure accurate response recording. Field notes were also recorded during and immediately following interviews to record contextual observations, nonverbal cues, and reflections on the data gathering process. During fieldwork, small changes were made to the interview strategy to increase clarity and flow; nonetheless, the primary subjects remained identical throughout all interviews. These judgements were recorded in field notes to ensure methodological transparency.

Data were collected using a semi-structured interview guide developed based on the study objectives and relevant literature on TB treatment adherence, social determinants of health, and health system barriers. The guide comprised open-ended questions designed to elicit participants’ experiences and perspectives, along with probing questions to explore emerging issues in greater depth. The semi-structured format ensured consistency across interviews while allowing flexibility to adapt to participants’ responses. The interview guide was reviewed by the research team to ensure content validity and clarity and was refined during the initial interviews to improve flow and comprehension.

The interview guide was pilot-tested with one participant, and minor revisions were made to improve question clarity; data from the pilot interview were not included in the final analysis.

### 2.4. Data Management

All interviews were audio-recorded and reviewed by the primary researcher to ensure completeness and accuracy of the data. The audio recordings were transcribed verbatim by the research team, with the primary researcher leading the transcription process. Interviews conducted in local languages were translated into English during transcription to maintain consistency in analysis. To ensure accuracy and credibility, all transcripts were cross-checked against the original audio recordings by members of the research team. Any discrepancies identified during this process were resolved through careful review of the recordings.

The audio recordings were transcribed verbatim. Transcripts from interviews conducted in local languages were translated into English to maintain consistency in analysis. All transcripts were checked against audio recordings to ensure accuracy. To maintain secrecy, all identifying information was stripped from transcripts, and participants were given unique codes. Digital data (audio files and transcripts) were securely stored on password-protected devices that only the research team could access.

### 2.5. Ethical Consideration

Ethical approval for the study was obtained from the Research Ethics and Biosafety Committee of the Faculty of Health Sciences, Walter Sisulu University (Reference: WSU HREC 178/2025), as well as the Eastern Cape Department of Health (Reference: EC_202507_038). Permission to conduct the study was obtained from facility managers prior to data collection. All participants provided written informed consent before participation. Participants were informed of their right to withdraw from the study at any time without any consequences. Confidentiality and anonymity were strictly maintained throughout the study. No identifying information is reported in this manuscript. No financial incentives were provided, and participation did not influence participants’ professional roles or responsibilities.

### 2.6. Data Analysis Procedure

Data analysis was conducted using thematic analysis, a widely used qualitative analytic method for identifying, analyzing, and reporting patterns within textual data. The analysis followed the six-phase approach proposed by Braun and Clarke (2006) [13], which allows for systematic organization and interpretation of qualitative data.

#### 2.6.1. Phase 1: Familiarization with the Data

The researcher began by listening to the audio recordings and reading the interview transcripts several times to gain a comprehensive understanding of the participants’ responses. During this stage, preliminary observations and reflections were recorded in field notes.

#### 2.6.2. Phase 2: Generating Initial Codes

Meaningful segments of the text related to TB treatment interruption were identified and labelled with descriptive codes. Coding involved systematically organizing the data into meaningful units that reflected key issues raised by the participants.

#### 2.6.3. Phase 3: Searching for Themes

Codes with similar meanings were grouped together to form broader categories. These categories were then organized into potential themes representing recurring patterns across the data.

#### 2.6.4. Phase 4: Reviewing Themes

The themes were reviewed and refined to ensure that they accurately represented the coded data and the overall dataset. At this stage, some themes were merged or redefined to ensure clarity and coherence.

#### 2.6.5. Phase 5: Defining and Naming Themes

Each theme was clearly defined and named to capture the essence of the participants’ perspectives. The themes were then organized in a logical structure to reflect the research objectives.

#### 2.6.6. Phase 6: Producing the Report

The final themes were presented, supported by direct quotations from participants to illustrate key findings. The analysis focused on understanding the factors contributing to TB treatment interruption and the strategies used by healthcare providers to address these challenges.

Although the initial coding was conducted by the primary researcher, the coding framework and emerging themes were reviewed collaboratively by members of the research team. Selected transcripts and coded data were independently examined by at least one additional researcher to enhance analytical rigor. Differences in coding and interpretation were discussed among the research team until consensus was reached. This iterative process strengthened the credibility, dependability, and confirmability of the analysis. An audit trail documenting coding decisions and theme development was maintained throughout the analysis process.

##### Coding Process

This coding process (Table 1) enabled the researcher to systematically organize the qualitative data and identify the major themes that emerged from participants’ experiences.

### 2.7. Quantitative Descriptive Analysis

Descriptive statistical analysis was used to summarise participant demographic and professional data, such as age and years of nursing experience. Furthermore, correlation and regression analyses were used to investigate the associations between variables. These analyses were conducted to give contextual understanding of the participant sample and to supplement the qualitative findings.

## 3. Results

### 3.1. Participant Demographic and Professional Characteristics

Participants (Table 2) had a mean age of 40.6 years (SD = 10.0; range 25–59), with a median age of 39 years, indicating that most participants were mid-career healthcare professionals. On average, participants had worked in the Nyandeni locality for 7.8 years (SD = 6.2; range 2–23) and reported a mean professional nursing experience of 14.2 years (SD = 9.3; range 2–32), reflecting substantial clinical exposure to TB management. A Pearson correlation analysis demonstrated a strong positive relationship between age and years of nursing experience (r = 0.89, *p* < 0.001), indicating that nursing experience increased significantly with age. A simple linear regression analysis further confirmed this relationship, showing that age was a significant predictor of nursing experience (*p* < 0.001) and explained 79% of the variance in experience (R^2^ = 0.79). The regression equation Experience = −19.37 + 0.83 (Age) suggests that each additional year of age was associated with an increase of approximately 0.83 years of nursing experience.

### 3.2. Facility Distribution Analysis

**Figure 1:**
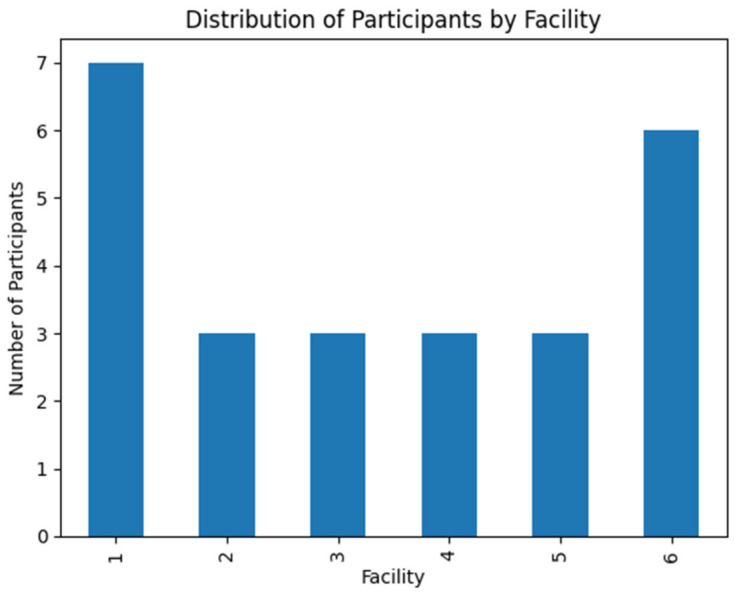
Distribution of participants by facility.

The distribution of participants across the six primary healthcare facilities in Figure 1 showed uneven representation. Facility 1 contributed the largest proportion of participants (28%), followed by Facility 6 (24%), while the remaining facilities each contributed approximately 12% of the sample. Despite this variation, the inclusion of participants from multiple facilities within the Nyandeni Subdistrict enhances the diversity of perspectives and ensures that the findings reflect experiences across different healthcare settings.

### 3.3. Distribution of Nursing Experience

There was a clear pattern indicating that older participants generally had more years of nursing experience, reflecting the expected progression of professional practice. Overall, the sample included a mix of early-career, mid-career, and highly experienced nurses across facilities, providing a diverse range of perspectives relevant to TB programme implementation.

### 3.4. Nursing Experience from Age

**Figure 2:**
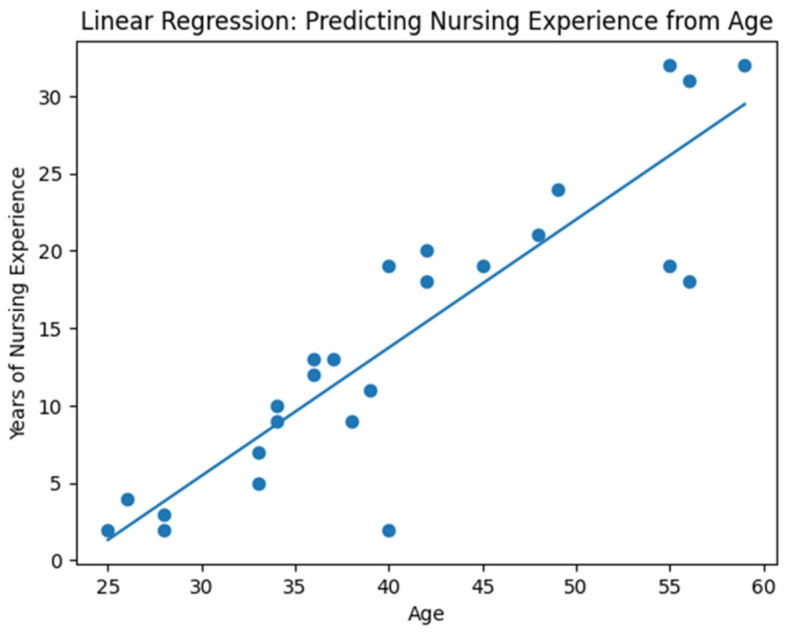
Regression Model Predicting Nursing Experience.

A linear regression analysis in Figure 2 was conducted to examine whether age predicts years of nursing experience among the participating professional nurses. The regression model produced the following equation: Years of Nursing Experience = −19.37 + 0.83 × Age.

The model demonstrated strong explanatory power, with an R^2^ value of 0.792, indicating that approximately 79% of the variation in nursing experience can be explained by age. The results suggest that for every one-year increase in age, nursing experience increases by approximately 0.83 years.

### 3.5. Cluster Analysis of Facility-Level Staffing Profiles

**Figure 3:**
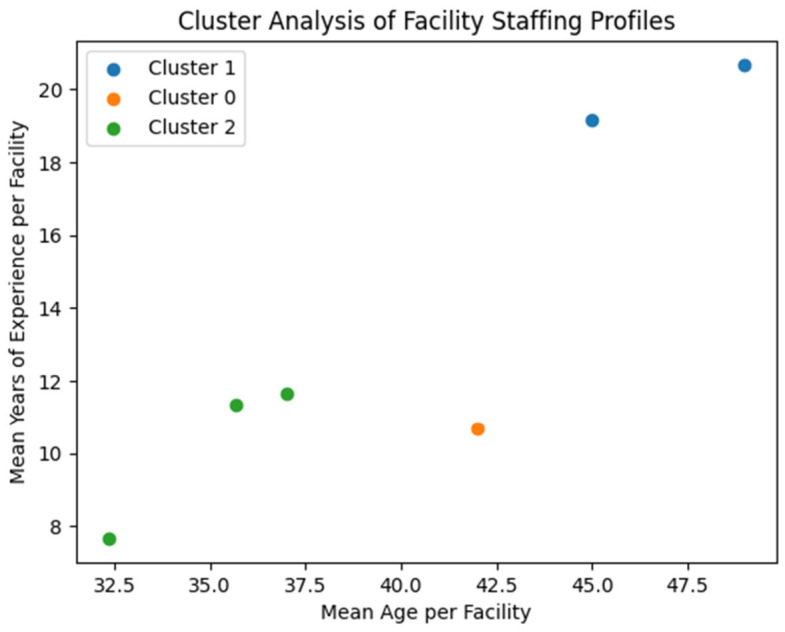
Cluster Analysis of Facility Staffing Profiles.

A cluster analysis in Figure 3 was conducted to determine whether the six participating facilities could be grouped according to staffing experience profiles, based on the mean age and mean years of nursing experience of nurses in each facility. The analysis identified three distinct facility clusters.

Cluster 1: Highly experienced facilities

Facilities 1 and 3 formed a cluster characterized by higher average staff age and the highest levels of nursing experience, with mean experience of approximately 20 years.

Cluster 0: Moderately experienced facility

Facility 2 formed an independent cluster characterized by a moderate age profile and moderate levels of professional nursing experience.

Cluster 2: Early- to mid-career workforce

Facilities 4, 5, and 6 formed a cluster characterized by younger staff profiles and lower average years of nursing experience compared with the other facilities.

The observed variation in staffing experience across facilities also provided important context for interpreting participants’ perspectives. Facilities with more experienced staff appeared to provide more system-level reflections on TB treatment interruption, including challenges related to patient tracing, long-term adherence, and structural socioeconomic barriers. In contrast, facilities with less experienced staff more frequently emphasised operational challenges, such as workload, patient follow-up difficulties, and day-to-day service delivery constraints.

### 3.6. Theme Co-Occurrence Network Diagram

**Figure 4:**
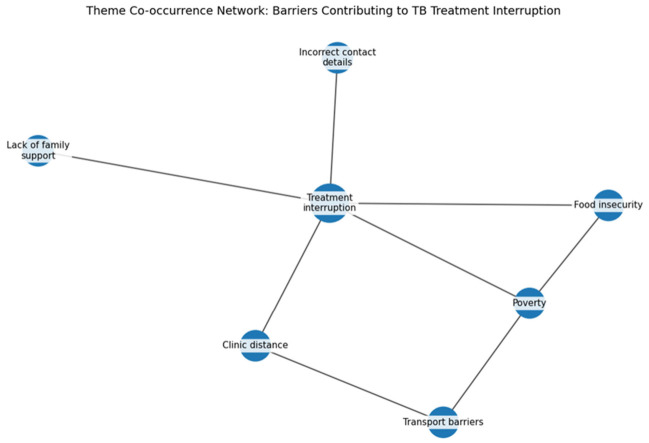
Network of Interacting Barriers.

The network diagram in Figure 4 illustrates the interconnected factors influencing TB treatment interruption as reported by participants. Treatment interruption emerged as the central node, linked to multiple barriers including poverty, food insecurity, transport barriers, clinic distance, lack of family support, and inaccurate patient contact details. The diagram further shows that poverty acts as a structural driver, directly linked to both food insecurity and transport barriers. These factors affect patients’ ability to attend clinic appointments and maintain consistent treatment adherence.

### 3.7. TB Treatment Interruption Conceptual Framework

**Figure 5:**
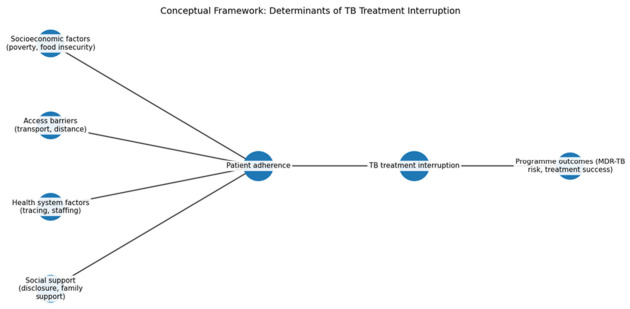
Conceptual Framework.

The framework presented in Figure 5 illustrates that TB treatment adherence is influenced by four main domains: socioeconomic factors (poverty and food insecurity), access barriers (long distances to clinics and transport costs), health system issues (patient tracing challenges and staffing shortages), and social support (family disclosure and household assistance). These determinants shape patient behaviors regarding treatment adherence, which if interrupted, can lead to serious consequences including increased multidrug-resistant tuberculosis (MDR-TB) risks and community transmission. Socioeconomic challenges impact patients’ ability to tolerate medication, while access barriers exacerbate adherence issues, especially in rural areas. Health system limitations hinder follow-up, and social support is crucial for ensuring treatment completion. Effective strategies must integrate approaches that address these interconnected factors to improve TB treatment outcomes.

These findings are supported by participants’ accounts, which highlight the interconnected nature of barriers to treatment adherence. One participant noted:

“Most patients stop treatment because they do not have food to take with the medication. When they feel weak, they just stop coming.”

(Participant 3)

Another explained:

“Patients live very far, and transport is expensive. If they don’t have money, they miss their appointments.”

(Participant 7)

### 3.8. TB Treatment Interruption Causal Pathway

**Figure 6:**
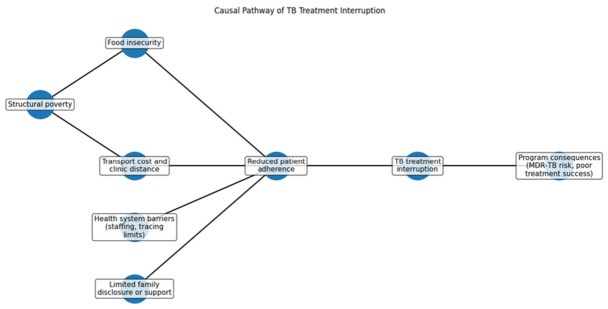
Causal Pathway Model.

Figure 6 outlines the complex interplay of structural, social, and health system factors affecting TB treatment adherence and the risk of treatment interruption. The model illustrates that TB treatment interruption arises from multiple interacting determinants rather than a single cause. Structural poverty contributes to food insecurity and transport barriers, particularly in rural areas, reducing patients’ ability to keep clinic appointments and adhere to treatment. Inadequate nutrition may also hinder medication tolerance, leading to treatment discontinuation. Health system constraints, such as limited patient tracing and staffing shortages, exacerbate these issues by preventing effective follow-up with non-adherent patients. Additionally, social factors, such as a lack of disclosure of TB status to family, can diminish emotional support vital for adherence. The cumulative effect of these factors can result in treatment interruption, leading to lower success rates and increased risk of multidrug-resistant tuberculosis, underscoring the need for comprehensive interventions addressing both social and health system barriers.

Participants described how multiple factors interact to influence treatment interruption:

“When a patient misses once, it becomes difficult to trace them, especially if their contact details are not correct.”

(Participant 5)

“If there is no support at home, the patient can easily default because no one is encouraging them to continue.”

(Participant 2)

### 3.9. Health System–Community Interaction Model for TB Adherence

**Figure 7:**
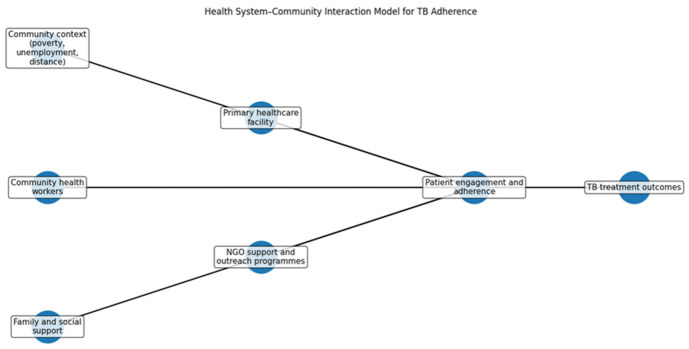
Health System–Community Interaction Model.

Figure 7 presents the multisectoral factors affecting patient engagement and adherence to tuberculosis (TB) treatment. It highlights the importance of the relationship between healthcare services and community support in promoting adherence. Socioeconomic factors, such as poverty and distance to healthcare facilities, impact patients’ access to care. Primary healthcare facilities provide essential services but community health workers (CHWs) play a vital role in linking patients with healthcare, offering education, and facilitating communication. Family support is crucial for adherence, as emotional and practical assistance significantly enhances treatment outcomes. The model asserts that strong collaboration between health systems and community support is essential for effective TB management and adherence monitoring.

### 3.10. TB Treatment Adherence Ecosystem Model

**Figure 8:**
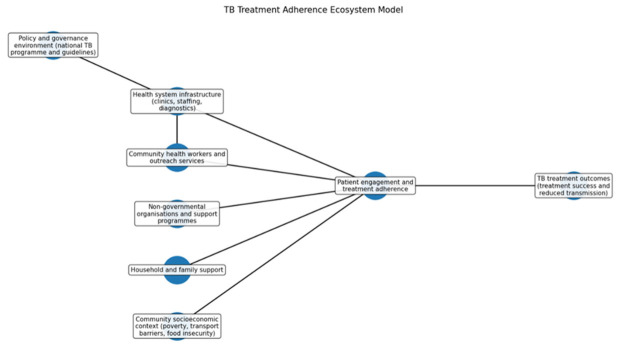
TB Treatment Adherence Ecosystem Model.

Figure 8 depicts the roles of policy, health systems, community structures, and household factors in influencing patient engagement and adherence to TB treatment. The TB treatment adherence ecosystem model illustrates adherence as an outcome of complex interactions across various levels: structural, health system, community, and household. Policies like the National TB Programme impact healthcare organization and resources, affecting primary healthcare access and staff. Primary healthcare facilities provide TB services, supported by community health workers and NGOs, which help facilitate adherence through patient tracing and education. Additionally, family support and socioeconomic factors like poverty and food insecurity affect patients’ access to healthcare and treatment compliance. The model highlights the necessity of coordinated actions across sectors for effective TB control, integrating policy, health system capacity, community networks, and household support.

### 3.11. Integrated Socio-Ecological Model of TB Treatment Interruption

**Figure 9:**
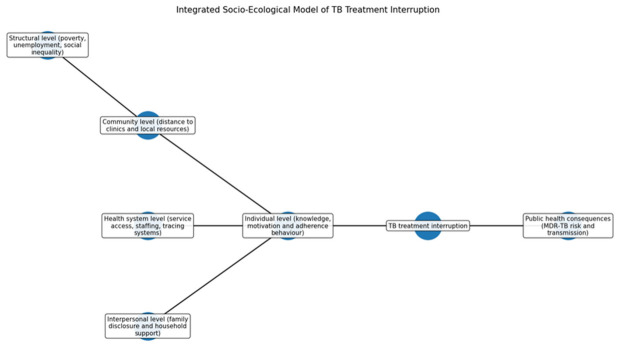
Socio-Ecological Model.

Figure 9 presents an integrated socio-ecological model that illustrates the multilevel determinants influencing TB treatment adherence, which can lead to treatment interruptions and public health issues. Structural factors like socioeconomic conditions create vulnerabilities in accessing healthcare, while community-level barriers, such as distance to facilities and local resource limitations, hinder clinic attendance. Health system elements, including staffing and service organization, directly impact adherence. Interpersonal factors, such as family support, and individual elements like patient knowledge and motivation also play critical roles. The interplay of these determinants necessitates multilevel interventions targeting structural inequalities, health system capacity, and social support to enhance TB treatment continuity and outcomes.

The role of community and health system interaction was emphasised by participants:

“Community health workers help us a lot because they follow up patients in their homes.”

(Participant 6)

“Sometimes we are short-staffed, so it becomes difficult to track all patients who miss appointments.”

(Participant 4)

## 4. Discussion

### 4.1. Participant Demographic and Professional Characteristics

The demographic and professional characteristics of the participating nurses provide important context for interpreting the findings of this study. Participants had a mean age of 40.6 years and an average of 14.2 years of nursing experience, indicating that most respondents were mid-career healthcare professionals with substantial clinical exposure to TB management. In rural healthcare settings, experienced nurses often serve as the backbone of primary healthcare systems, particularly in TB-endemic regions where nurses play a central role in diagnosis, treatment initiation, patient education, and adherence monitoring. Previous studies have similarly reported that primary healthcare nurses in high TB burden settings often possess extensive clinical experience due to the decentralization of TB services to primary care facilities [14,15].

The average duration of employment within the Nyandeni locality was 7.8 years, suggesting that many participants had developed familiarity with local community dynamics and healthcare system challenges. Local experience is particularly important for TB programme implementation because healthcare providers who understand community-specific socioeconomic barriers may be better positioned to identify patients at risk of treatment interruption and implement targeted adherence support strategies. Studies conducted in rural African healthcare systems have shown that healthcare workers with strong contextual knowledge of local communities can significantly improve patient follow-up and treatment adherence outcomes [16].

### 4.2. Distribution of Nursing Experience Across Facilities

The analysis of nursing experience across facilities revealed variation in workforce composition within the Nyandeni Subdistrict. Facilities 1 and 3 were characterized by highly experienced staff, whereas Facility 5 had comparatively lower median experience levels. Such variation in workforce experience may have important implications for TB programme implementation and service delivery.

Facilities with more experienced staff may benefit from stronger clinical leadership and institutional knowledge regarding TB programme management. Experienced nurses are often responsible for coordinating TB services, mentoring junior staff, and managing complex cases such as treatment complications or treatment interruption. Previous research has shown that workforce experience can influence the quality of clinical decision-making and adherence monitoring in TB programmes [17,18].

Conversely, facilities with younger or less experienced staff may face greater operational challenges, particularly in resource-limited settings where staff must manage multiple clinical responsibilities. Workforce turnover and uneven distribution of experienced staff have been identified as important health system barriers affecting TB programme performance in several African countries [19,20,21]. Strengthening mentorship systems and continuous professional development opportunities may therefore be essential for improving TB programme implementation across facilities.

### 4.3. Relationship Between Age and Nursing Experience

The observed relationship between age and nursing experience reflects the expected progression of professional practice within the healthcare workforce. Importantly, the inclusion of participants across different experience levels strengthens the study by capturing a diversity of perspectives on TB treatment interruption. More experienced nurses often provided insights into systemic and long-term challenges affecting adherence, while less experienced nurses highlighted operational and day-to-day barriers encountered in TB programme implementation. This finding is consistent with workforce analyses conducted in other primary healthcare settings, where age and professional experience have been shown to be closely associated [22]. The presence of participants across multiple career stages strengthens the credibility of the study findings because it ensures that perspectives were obtained from both early-career nurses and highly experienced practitioners.

In the context of TB programme implementation, professional experience can play an important role in identifying patients at risk of treatment interruption and managing complex treatment regimens. Experienced healthcare providers may be better equipped to detect early signs of non-adherence, manage adverse drug reactions, and provide patient counselling during long treatment courses.

### 4.4. Nursing Experience from Age

Given the strong association between age and experience, age may serve as a proxy for clinical exposure in this setting. However, rather than being a primary analytical focus, this relationship is relevant in understanding the range of perspectives contributing to the findings. From a health system perspective, these findings suggest that workforce age profiles may provide useful insight into the distribution of clinical experience within healthcare facilities. Maintaining a balanced workforce composed of both experienced and early-career healthcare workers is important for ensuring knowledge transfer and mentorship within clinical teams. Previous studies have highlighted the importance of mentorship and supportive supervision in strengthening TB programme performance and improving treatment adherence monitoring [23].

### 4.5. Facility-Level Staffing Profiles

Differences in staffing profiles across facilities provide important insight into how TB treatment interruption is perceived and managed. Facilities with more experienced staff appeared to demonstrate greater awareness of structural and systemic challenges, such as socioeconomic barriers and patient tracing limitations. In contrast, facilities with less experienced staff more frequently emphasised operational challenges, including workload pressures and follow-up difficulties. This suggests that workforce experience may influence not only service delivery capacity but also how barriers to treatment adherence are identified and addressed. These findings are consistent with research indicating that health workforce distribution plays a significant role in the effectiveness of TB control programmes. In many rural health systems, shortages of experienced healthcare workers can limit the capacity of facilities to provide comprehensive adherence monitoring and patient follow-up [24]. Ensuring equitable distribution of experienced staff across facilities may therefore be an important strategy for strengthening TB programme performance.

The variation in staffing profiles across facilities may also help explain differences in how determinants of TB treatment interruption were perceived and articulated. Facilities with more experienced nurses may be better positioned to recognise broader structural and health system challenges affecting adherence, whereas less experienced staff may focus more on immediate operational barriers. This highlights the importance of workforce experience not only for service delivery but also for shaping how challenges within TB programmes are identified and addressed.

### 4.6. Interconnected Determinants of TB Treatment Interruption

The thematic network analysis highlights the complex and interconnected nature of factors contributing to TB treatment interruption. Rather than being driven by a single determinant, treatment interruption appears to arise from the interaction of multiple socioeconomic, health system, and social support barriers.

Poverty emerged as a central structural determinant linking several other barriers, including food insecurity and transport challenges. These findings are consistent with previous studies demonstrating that socioeconomic vulnerability significantly affects TB treatment adherence in resource-limited settings [25]. Patients experiencing poverty may struggle to attend clinic appointments or tolerate medication without adequate nutrition, particularly when TB drugs must be taken with food to minimize adverse side effects.

Transport barriers and long distances to healthcare facilities further exacerbate these challenges. Geographic access to healthcare services remains a significant barrier in many rural regions of sub-Saharan Africa, where patients may need to travel long distances to reach primary healthcare facilities [26]. Transport costs can therefore represent a major obstacle to consistent treatment adherence.

Health system factors also contribute to treatment interruption. Participants highlighted challenges related to patient tracing and staffing limitations, which may weaken follow-up mechanisms designed to support patients who miss appointments. Previous studies have demonstrated that community health worker programmes play a critical role in tracing patients and improving TB treatment adherence [27].

Social support structures also influence treatment adherence. Patients who lack family support or who have not disclosed their TB status may experience greater difficulties completing treatment. Research has consistently shown that family support, treatment literacy, and reduced stigma are important facilitators of TB treatment adherence [28].

Overall, these findings reinforce the growing recognition that TB treatment interruption is a multilevel phenomenon influenced by structural, social, and health system determinants. Addressing treatment interruption therefore requires integrated interventions that combine social protection measures, improved healthcare access, strengthened patient tracing systems, and enhanced community-based support mechanisms.

## 5. Study Limitations

Despite the strengths of this study, several limitations should be acknowledged.

First, the study was conducted in six primary healthcare facilities within the Nyandeni Subdistrict, which may limit the generalizability of the findings to other districts or provinces. However, qualitative research aims to provide in-depth contextual understanding rather than statistical generalization.

Second, the study relied on self-reported experiences and perceptions of professional nurses, which may be influenced by personal perspectives or recall bias. Nevertheless, participants were directly involved in TB programme implementation and therefore provided valuable insights based on their professional experience.

Third, the study focused exclusively on healthcare providers’ perspectives and did not include the views of TB patients who had interrupted treatment. Including patients’ perspectives could provide a more comprehensive understanding of the factors influencing treatment interruption.

Finally, time constraints and workload pressures within healthcare facilities may have influenced the availability of participants for interviews. However, efforts were made to schedule interviews at convenient times to minimize disruption to clinical services.

Despite these limitations, the study provides valuable insights into the challenges experienced by healthcare providers in managing TB treatment interruption in rural healthcare settings and highlights opportunities for strengthening TB adherence interventions.

## 6. Novel Contributions and Context-Specific Implications

While the determinants of TB treatment interruption identified in this study such as poverty, food insecurity, transport barriers, and limited social support have been reported in the previous literature, the present study offers important novel contributions.

First, this study provides context-specific insights from rural primary healthcare settings in the Eastern Cape, where structural inequalities, geographic barriers, and health system constraints interact in unique ways. Unlike studies based on secondary data or literature reviews, the findings presented here are grounded in the lived experiences of frontline healthcare providers, who directly manage TB patients within resource-limited environments.

Second, the study advances existing knowledge by illustrating how these determinants interact dynamically across multiple levels, rather than acting as isolated factors. The conceptual models developed from participant narratives demonstrate how structural poverty influences food insecurity and transport access, which in turn affects clinic attendance and treatment adherence. These multilevel interactions are often underrepresented in conventional analyses.

Third, the findings highlight health system realities that are not always captured in the literature, including challenges related to patient tracing, staffing constraints, and operational pressures within primary healthcare facilities. These insights underscore the value of qualitative inquiry in uncovering system-level barriers that may not be evident in quantitative or review-based studies.

Importantly, this study provides actionable, context-specific implementation insights for TB programmes in South Africa. The findings suggest that improving treatment adherence requires integrated interventions that combine:

- Strengthened community health worker programmes for patient tracing,
- Targeted social support interventions addressing food insecurity and transport barriers,
- Decentralised and accessible TB services
- Strengthening facility-level capacity and workforce support.

These findings reinforce the need for locally tailored, multisectoral approaches to TB control that are responsive to the realities of rural healthcare settings, rather than relying solely on generalised strategies derived from broader literature.

## 7. Recommendations for Future Research

While this study provides valuable insights into healthcare providers’ perspectives on TB treatment interruption, several areas require further investigation.

First, future research should include the perspectives of TB patients who have experienced treatment interruption. Understanding patients’ lived experiences would provide a more comprehensive understanding of the social, economic, and behavioral factors influencing adherence.

Second, quantitative studies examining the relative contribution of different determinants of treatment interruption could help inform targeted interventions. Larger studies across multiple districts could also assess whether the patterns observed in this study are consistent in other rural settings.

Third, implementation research evaluating the effectiveness of community-based adherence interventions would be valuable. Interventions such as community health worker support programmes, digital adherence technologies, and nutritional support initiatives should be assessed to determine their impact on improving treatment adherence.

Fourth, research exploring health system factors affecting TB programme implementation, including staffing levels, training, and patient tracing systems, could help identify operational improvements needed to strengthen TB control efforts.

Finally, longitudinal studies examining treatment outcomes over time may provide deeper insights into how structural and health system factors interact to influence TB treatment success.

## 8. Conclusions

This study explored the factors influencing TB treatment interruption from the perspectives of professional nurses working in primary healthcare facilities in the Nyandeni Subdistrict. The findings demonstrate that TB treatment interruption is a complex phenomenon influenced by interacting socioeconomic, health system, and social support factors.

Key barriers identified in the study include poverty, food insecurity, transport difficulties, long distances to healthcare facilities, limited family support, and challenges related to patient tracing. These factors collectively influence patients’ ability to maintain treatment adherence throughout the TB treatment course.

The conceptual frameworks developed in this study illustrate that TB treatment interruption arises from multilevel interactions between structural conditions, community contexts, health system capacity, interpersonal relationships, and individual behaviours. Addressing treatment interruption therefore requires comprehensive interventions that extend beyond clinical care and incorporate social support and community engagement strategies.

Strengthening community health worker programmes, improving patient tracing systems, expanding access to healthcare services, and addressing socioeconomic barriers may significantly improve treatment adherence and TB programme outcomes in rural settings.

Ultimately, improving TB treatment adherence is essential for reducing treatment failure, preventing the emergence of multidrug-resistant tuberculosis, and limiting ongoing transmission within communities. A coordinated approach involving healthcare systems, communities, and policymakers is therefore critical for strengthening TB control efforts and achieving sustainable improvements in public health outcomes.

## Figures and Tables

**Table 1:** Presents how raw interview data were coded and organized into themes during the analysis process.

Participant Quote	Code	Category	Theme
“Most patients stop treatment because they do not have food to take with the medication.”	Lack of food	Socioeconomic barriers	Challenges in managing treatment interruption
“Some patients live very far from the clinic and cannot afford transport.”	Transport difficulties	Access barriers	Challenges in managing treatment interruption
“We normally trace patients using community health workers when they miss appointments.”	Patient tracing	TB management strategies	Measures used to manage treatment interruption
“If patients stop treatment, they may develop drug-resistant TB.”	Risk of drug resistance	Consequences of interruption	Effects of TB treatment interruption

**Table 2:** Descriptive and inferential statistical summary of participant demographic and professional characteristics.

Analysis	Variables	Statistical Test	Result	Interpretation
Descriptive statistics	Age	Mean ± SD	40.6 ± 10.0 years	Mid-career workforce
Descriptive statistics	Years working in locality	Mean ± SD	7.8 ± 6.2 years	Mixed local experience
Descriptive statistics	Nursing experience	Mean ± SD	14.2 ± 9.3 years	Substantial clinical experience
Correlation analysis	Age vs. nursing experience	Pearson correlation	r = 0.89, *p* < 0.001	Strong positive relationship
Regression analysis	Age → experience	Linear regression	R^2^ = 0.79, *p* < 0.001	Age strongly predicts experience
Cluster analysis	Facility staffing profiles	K-means clustering	3 clusters identified	Facilities differ in workforce experience

## Data Availability

Data can be requested from the corresponding author.

## Abbreviations

The following abbreviations are used in this manuscript:

MDR-TB	Multidrug resistant tuberculosis
TB	Tuberculosis
WHO	World Health Organization
